# The proBNPage reduction (PBAR) trial—results of a randomized, double blind, placebo-controlled, pilot study to fine tune an NT-proBNP-based method to assess the effect of anti-aging treatments

**DOI:** 10.1007/s11357-025-01827-y

**Published:** 2025-09-08

**Authors:** Antonio Muscari, Paola Forti, Donatella Magalotti, Mara Brizi, Filomena Piro, Eric Ramazzotti, Loredana Incorvaia, Paolo Maltoni, Daniele Casanova Borca, Eleonora Capelli, Barbara Ciuffi, Paolo Pandolfi, Giovanni Barbara

**Affiliations:** 1 Department of Medical and Surgical Sciences, University of Bologna, Via Massarenti, 9–40138, Bologna, Italy; 2 Medical-Surgical Department of Digestive, Hepatic and Endocrine-Metabolic Diseases, IRCCS Azienda Ospedaliero-Universitaria di Bologna, Bologna, Italy; 3 Pharmaceutical Department, IRCCS Azienda Ospedaliero-Universitaria di Bologna, Bologna, Italy; 4 LUM Metropolitan Laboratory, AUSL Bologna, Bologna, Italy; 5 Laboratorio Unico Della Romagna, AUSL Romagna, Pievesestina di Cesena, Italy; 6 Epidemiological and Health Promotion Unit, Department of Public Health, AUSL Bologna, Bologna, Italy

**Keywords:** Biological age, Coenzyme Q10, NT-proBNP, Resveratrol, Selenium, TA-65

## Abstract

NT-proBNP levels increase exponentially with age and are associated with cardiovascular and all-cause mortality. From NT-proBNP concentration a surrogate for biological age (“proBNPage”) can be obtained. The primary objective of this study was to define a method to design future trials on anti-aging treatments using proBNPage. The secondary objective was the tentative evaluation of 4 potential anti-aging dietary supplements. The trial lasted 2 years and involved 120 healthy subjects aged 71.7 ± 4.6 years divided into 3 groups: A) coenzyme Q10 100 mg bid + selenium 100 mcg, B) resveratrol 350 mg bid + TA-65 100U, C) placebo. ProBNPage was the primary outcome variable. Secondary variables included step test duration, handgrip strength, and self-reported health status. In group B, the study was interrupted at month 5 due to significant LDL-cholesterol increase, probably caused by the high-dose resveratrol. Partly because of dropouts and inadequate treatment intake, groups A and C showed non-significant differences in the primary and secondary variables. Therefore, these groups were considered a single group, in which after 2 years proBNPage increased by 2.5 years (*P* = 0.01), particularly in subjects over 70 (+ 3.0 years, *P* = 0.009). It was thus calculated that 126 subjects over 70 could allow the assessment of a 2-year treatment vs. placebo with a power of 80%. This study has developed an NT-proBNP-based method to evaluate future interventions on biological age. The treatments did not induce significant changes in proBNPage. The marked increase in cholesterol in group B suggests that high-dose resveratrol should be avoided.

**Supplementary Information:**

The online version contains supplementary material available at 10.1007/s11357-025-01827-y.

## Introduction

Age is the main risk factor for many diseases but, unfortunately, it is a non-modifiable risk factor. On the other hand, the observation that not all people age in the same way has led to the definition of the concept of biological age [1] which, unlike chronological age, could perhaps be modified. In recent years, several methods have been proposed to quantify biological age, mainly based on the determination of biological processes associated with aging (such as DNA methylation [2] and telomere attrition [3]), or on the evaluation of indices composed of multiple clinical or laboratory biomarkers [4, 5]. The former are very reliable, but are only within the reach of a few laboratories, while the latter are more suitable for defining people’s health status, rather than their biological age. These drawbacks have so far hindered the research into possible interventions to slow down the progression of biological age in humans, when, on the other hand, at the experimental level numerous substances potentially useful in this sense have been identified [6].

The N-terminal fragment of the B-type natriuretic peptide precursor (NT-proBNP) can be easily measured in most laboratories for the diagnosis of heart failure. It is released by cardiomyocytes subjected to mechanical or ischemic stress, and its blood concentrations increase exponentially with advancing age, probably because cardiomyocytes are progressively reduced in number and are therefore subject to increasing tension. Thus, the relationship between NT-proBNP and age seems to be due to aging-related processes like sarcopenia, apoptosis, and cellular senescence. The correlation with age is very strong, and higher than that of other laboratory variables [7, 8]. In addition, NT-proBNP is associated with cardiovascular and all-cause mortality even more strongly than chronological age [7, 9].

In consideration of these characteristics, we have proposed NT-proBNP as a possible marker of biological age [7], also highlighting its relationships with morbidity and with several cardiovascular, physical, and psychological aging-related manifestations [10]. After logarithmic transformation of the concentrations, the relationship of NT-proBNP with age becomes linear. Knowing the parameters of this linear regression, starting from the concentration of NT-proBNP, it is possible to calculate the average age associated with it; the “proBNPage,” which unlike NT-proBNP, has a Gaussian distribution [7].

Perhaps the most relevant aspect of biological age determination is its possible use to establish the effectiveness of potential anti-aging treatments. In particular, in a longitudinal study, it could be verified whether the progression of biological age in the treated group is slowed, stopped, or even reversed compared to the progression of biological age in the placebo group. In an attempt to apply this idea to proBNPage, taking into account the information derived from a previous cross-sectional study [9, 11], we designed a trial (ProBNPage reduction–PBAR trial) [12] based on these hypotheses: (1) that proBNPage may have a progressive increase as time goes by, up to become statistically significant in paired tests compared to baseline; (2) that a relatively limited observation period (2 years) and small sample sizes, thanks to the power of paired tests, may be sufficient to demonstrate differences in behavior between a treated group and a placebo group. The primary objective of the PBAR trial was to describe the spontaneous longitudinal course of proBNPage, in order to find the parameters needed to design future studies on anti-aging treatments. The secondary objective was to ascertain whether some dietary supplements, which had been shown to be promising in previous studies, could promote a non-significant increase, or even a halt in the progression, of proBNPage.

The trial (randomized, double-blind, and placebo-controlled) included these three treatment groups: (A) coenzyme Q10 + selenium; (B) resveratrol + TA-65; (C) placebo. After selection and randomization, the treatment intake began. In the safety checks carried out at the 3rd month, a significant increase in blood cholesterol was detected in the entire sample. To establish whether this increase was generalized or instead concerned only one of the three groups, the Clinical Trials Center of our hospital commissioned a statistical expert from outside the group of investigators to verify the trend of cholesterol within the three treatment groups. It was thus ascertained that the increase selectively concerned group B. The Clinical Trials Center therefore decided to stop the study in group B at the 5th month and to continue the trial in groups A and C, maintaining the double-blind mechanism for the investigators.

This report outlines the main findings of the PBAR trial and the main conclusions we drew from it.

## Methods

### Participants

This study was approved by the Ethics Committee of the Emilia-Romagna Region Area Vasta (CE-AVEC) on March 16, 2022 (ID: 64/2022/Sper/AOUBo). Trial registration: ClinicalTrials.gov (US NIH), ID: NCT05500742, August 19, 2022.

**Fig. 1:**
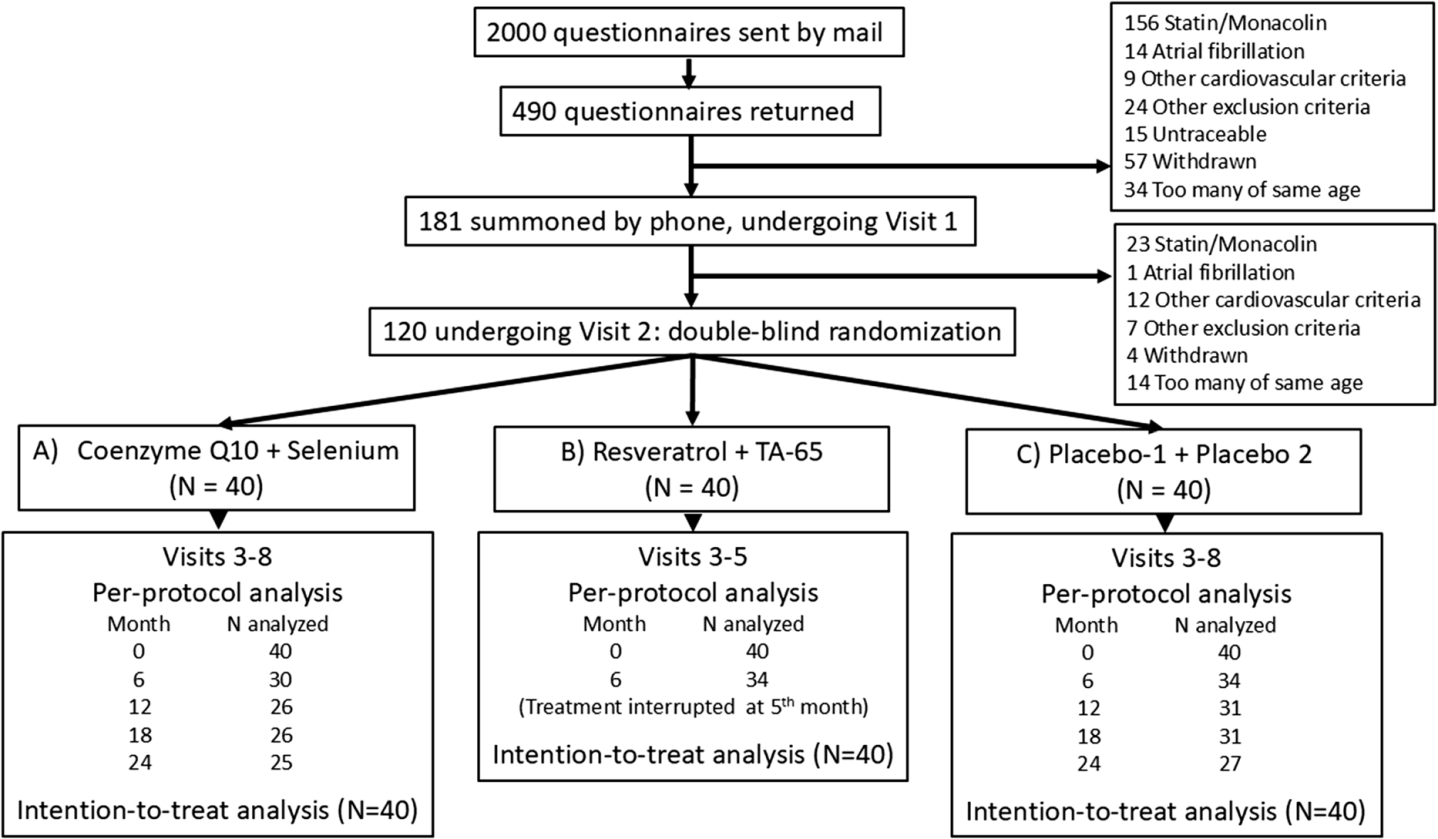
Schematic flow-chart of PBAR trial

The PBAR trial protocol has been described in detail elsewhere [12]. In brief (Fig. 1), the local Department of Public Health identified the names and addresses of 2000 subjects, 1000 men and 1000 women, aged between 65 and 80 years, residing near the S. Orsola-Malpighi Hospital in Bologna, who were apparently healthy (no hospitalization in the last 3 years and intake of a maximum of four drugs per day). In July 2022, each of these subjects was sent a letter of invitation and a questionnaire to be completed, with a pre-paid return envelope, concerning demographic data, risk factors, previous diseases, and current health status. As expected, the acceptance rate was about 25% and 490 completed questionnaires were returned. Based on the information obtained with the questionnaires, 203 subjects were excluded because of exclusion criteria (the main ones were cardiovascular diseases, atrial fibrillation, other serious diseases and taking statins or monacolin K present in red rice supplements) (see Supplementary Table 1 for the complete list of inclusion and exclusion criteria). Statin intake was the most frequent exclusion criterion (156 subjects). In fact, statins can interfere with Coenzyme Q10 [13] and can activate telomerase [14] similarly to TA-65. Another 106 subjects were excluded as they could not be traced by telephone or withdrew spontaneously or were too numerous in the same age group. At the end of this selection, 181 subjects remained, who were invited by telephone to present themselves at the first visit. During visit 1 (screening assessment), after providing written informed consent, participants underwent various investigations (medical examination, ECG, routine laboratory tests, evaluation of previous documentation) which led to the exclusion of another 61 subjects. At the end of these selection phases, 120 participants, 60 men and 60 women, remained and during visit 2 (baseline assessment, November 2022) they were randomized into the three treatment groups (40/group) with a procedure stratified according to age and sex. In this way, each of the three groups included 20 men (10 above and 10 below the median age) and 20 women (10 above and 10 below the median age), thus making it unlikely that random differences in age and sex could occur among the groups. During the visits, outcome and safety variables were assessed as follows: visit 2 (baseline)—outcome + safety; visit 3 (14 days)—safety; visit 4 (3 months)—safety; visit 5 (6 months)—outcome + safety (serum lipids only); visit 6 (12 months)—outcome + safety; visit 7 (18 months)—outcome; visit 8 (24 months)—outcome + safety. The last patient was seen on November 18, 2024.

### Outcome variables

ProBNPage was the primary outcome variable. It was obtained from the blood concentration of NT-proBNP through these two formulas (7):

- proBNPage_men_ = [ln(NT-proBNP) + 1.2958]/0.0827
- proBNPage_women_ = [ln(NT-proBNP) − 1.5258]/0.0478

NT-proBNP measurements were performed on the same day of the visits, in whole blood, by a point-of-care fluorescence immune assay (Quidel Triage NT-proBNP, Quidel Cardiovascular Inc., San Diego, USA). High NT-proBNP values allowed us to identify new cases of paroxysmal atrial fibrillation, which were then excluded from the study. For these measurements during the 2 years of study, nine different batches of reagents were used, with a coefficient of variation of 16%. In order to reduce as much as possible the variability due to technical factors, at the end of the study, all measurements were repeated on frozen serum aliquots by means of an electrochemiluminescence immunoassay (proBNP Elecsys, Roche Diagnostics, Mannheim, Germany). For these serum assays, a single batch of reagents with a coefficient of variation of 5% was used, and the concentrations thus determined were included in the formulas for calculating the proBNPage.

The secondary outcome variables were as follows: (1) time in seconds to perform the step test, a fitness indicator associated with aerobic capacity [15], which consists of going up and down 2 steps 20 times; (2) handgrip strength in kg, which was determined by a digital dynamometer; (3) self-assessment of well-being/health status by a score ranging between 0 and 100, which was carried out using the Visual Analog Scale (VAS) present in the EuroQoL 5D questionnaire [16].

### Safety variables

The safety of the treatments was assessed with the periodic determination of clinical, instrumental, and laboratory variables: body weight, blood pressure, pulse oximetry, ECG, complete blood count, total cholesterol, HDL cholesterol, LDL cholesterol, triglycerides, creatinine, Na +, K +, Ca + +, AST, ALT, gammaGT, creatine kinase, erythrocyte sedimentation rate (ESR), C-reactive protein (CRP), blood glucose, glycated hemoglobin (HbA1c), INR, aPTT, and (in men) total PSA.

After each control session, safety variables were compared with baseline values in the whole sample by paired tests, to detect possible highly significant (*P* < 0.001) and clinically relevant differences. This in fact happened at the 3rd month, when total cholesterol was found to be increased from 214.8 ± 29.5 to 228.2 ± 35.2 mg/dl (*P* < 0.0001), a 6.2% increase during 3 months of treatment. A report was then sent to both the Clinical Trial Center of our hospital and the Ethics Committee, which subsequently led to the discontinuation of group B.

### Treatments

Every 6 months, participants received a sealed bag containing two boxes (treatment 1 and treatment 2), each containing enough capsules for 6 months. Patients had to take treatment 1 twice a day (morning and evening) and treatment 2 once a day (evening). Bags and boxes were prepared and numbered from 1 to 120 by the hospital pharmacy according to a randomization list set up by the Research and Innovation Unit of our hospital. From visit 5 onwards, participants returned the boxes with any remaining capsules, which were counted at the hospital pharmacy. Subjects who had returned 60 or more capsules of treatment 1 or 30 or more capsules of treatment 2 were excluded from the 6-month analysis. Neither the investigators nor the participants knew the contents of the boxes. The double-blind mechanism was opened only after the database was closed.

- *Group A* was treated with coenzyme Q10 100 mg bid + selenium 100 mcg (Q10 Gold and SelenoPrecise, Pharma Nord ApS, Vejle, Denmark).

Coenzyme Q10 is a powerful antioxidant that exerts important functions in cellular respiration and energy production. It reduced cardiovascular and total mortality in patients with heart failure [17]. Its supplementation is virtually free of harmful effects. Selenium and selenoproteins also contribute to our body’s antioxidant systems. Blood selenium levels and dietary selenium intake are inversely associated with mortality [18, 19]. On the other hand, administration of selenium in high doses (> 300 mcg/day) resulted in increased mortality [20]. In Italy, the maximum dose allowed is 100 mcg/day. Administration of coenzyme Q10 at a daily dose of 100 mg bid for 4 years, together with selenium 200 mcg, reduced cardiovascular mortality in elderly adults [21], and this effect was maintained up to 12 years after the trial [22]. To the best of our knowledge, no other combination of supplements has resulted in similar effects on an unselected sample of the elderly population. More recently, the same association has been reported to prevent telomere attrition in leukocytes [23].

- *Group B* was treated with resveratrol 350 mg bid (NOW Foods, Bloomingdale, IL, USA) + TA-65 100 U (T.A. Sciences, New York, NY, USA).

Resveratrol is a powerful polyphenolic antioxidant found in small amounts in the skins and seeds of grapes and in the roots of *Polygonum cuspidatum*. It exhibits anti-inflammatory effects [24], and also appears to activate telomerase [25] and inhibit the TOR system [26]. Resveratrol prolonged life in drosophilas, roundworms, killifish, and obese mice [27, 28]. In humans, resveratrol exhibited beneficial effects on diabetes, dyslipidemia, and blood pressure, and perhaps even an anti-cancer action [29, 30], but so far, no study has documented effects on human longevity. The bioavailability of resveratrol is very low, due to significant intestinal and hepatic inactivation [31]. Prior to the start of this study, resveratrol doses above 1000 mg/day were known to be associated with an increased incidence of gastrointestinal adverse events [32]. A hypercholesterolemic effect was subsequently reported for doses above 500 mg/day [33], which also occurred in our group B.

TA-65 is a derivative of *Astragalus membranaceus* root. In humans, TA-65 has been shown to lengthen telomeres in chromosomes [34], an effect that has the potential to increase longevity. In addition, in humans, TA-65 improved age-related macular degeneration [35] and some blood parameters associated with the metabolic syndrome [36]. TA-65 enhanced immunity and reduced inflammation in post-myocardial infarction patients [37]. At a dose of 100 U/day, TA-65 reduced the number of senescent T cells in healthy volunteers [38]. No significant adverse effects of this supplement have been reported in clinical studies, and it has been awarded the GRAS (Generally Recognized As Safe) certification.

- *Group C* was treated with placebo 1 bid + placebo 2 (both provided by Pharma Nord ApS, Vejle, Denmark).

### Statistical analysis

The variables were described with mean and standard deviation or 95% confidence interval (C.I.) in case of normal distribution, or with median and interquartile range in case of non-Gaussian distribution. Similarly, the differences among groups were tested by one-way ANOVA or by Kruskal–Wallis test, as appropriate. The differences among percentages were tested by chi-square test. For adverse events, incidence rates per 100 person-months were reported and compared by rate ratio test. Pearson’s correlation coefficients were determined by linear regression after logarithmic transformation of the log-normal variables. The effect of treatments on outcome variables was assessed by three different procedures: per-protocol, intention to treat, and linear mixed models. Changes at each semester compared to baseline (within-group differences) were assessed by paired *t*-tests or Wilcoxon’s test, as appropriate. Changes in proBNPage between groups were assessed by ANOVA for repeated measures. The intention-to-treat analysis was performed after multiple imputation for missing values of proBNPage. In addition, a sensitivity analysis was performed, by imputation of the highest or lowest detected values in the place of missing values, in order to promote the best or worst possible result for treatments. Finally, linear mixed models were applied to primary and secondary outcome variables including all available observations, also accounting for the effect of covariates. Linear mixed models were also used to evaluate the possible influence of confounding factors on proBNPage across the longitudinal measurements. Sample size estimations were carried out using G*Power software v. 3.1 (*F* tests, ANOVA: repeated measures). The statistical analysis was carried out by R-based software (Jamovi v. 2.6.17).

Two-tailed tests were performed throughout. *P* values < 0.05 were considered significant and Holm-Bonferroni correction was applied in case of multiple testing.

## Results

Considering the baseline sample as a whole (*N* = 120), the mean age was 71.7 ± 4.6 years (72.6 ± 4.6 in males and 70.8 ± 4.4 in females, *P* = 0.03), while the mean proBNPage was 66.5 ± 10.5 years (64.9 ± 9.1 in males and 68.1 ± 11.5 in females, *P* = 0.10). Table 1 shows the baseline characteristics of the three groups, which did not differ significantly for any of the variables considered. NT-proBNP and the corresponding proBNPage were lower in group C than in the other two groups, but these differences were not significant. The secondary outcome variables (step test duration, handgrip strength, and EuroQoL VAS) at baseline also did not differ significantly among the three groups.

**Table 1:** Baseline characteristics of the 3 groups

Variable	Group C (Placebo)	Group A (Coenzyme Q10 + Selenium)	Group B (Resveratrol + TA-65)	*P *value
	*N* = 40	*N* = 40	*N* = 40	
Age (years)	71.9 ± 4.0	71.4 ± 5.3	71.9 ± 4.3	0.87
Male sex	20 (50)	20 (50)	20 (50)	-
ProBNPage (years)	68.3 ± 11.6	66.3 ± 9.5	65.1 ± 10.2	0.43
NT-proBNP (pg/ml)	110.0 [68.5–167.3]	110.0 [51.2–136.5]	81.6 [43.6–122.8]	0.20
Education (years)	17 [13–17]	13 [13–17]	13 [8–17]	0.67
Hypertension	18 (45.0)	11 (27.5)	16 (40.0)	0.25
Hypercholesterolemia	12 (30.0)	14 (35.0)	11 (27.5)	0.76
Ever smoker	25 (62.5)	19 (47.5)	21 (52.5)	0.39
Alcohol > 2 units/day	7 (17.5)	4 (10.0)	4 (10.0)	0.50
BMI (kg/m^2^)	26.2 ± 4.0	26.0 ± 4.6	26.7 ± 4.5	0.74
PASE	80.1 [56.2–109.1]	68.4 [38.9–102.0]	71.2 [53.2–92.5]	0.50
Step test (s)	94.6 ± 25.3	95.5 ± 24.9	97.3 ± 19.3	0.86
Handgrip strength (kg)	34.1 ± 10.2	31.8 ± 8.9	33.1 ± 11.2	0.54
EuroQoL VAS	80.5 ± 10.2	77.4 ± 11.2	78.3 ± 8.9	0.40
Heart rate (beats/min)	68.2 ± 11.8	65.8 ± 11.1	67.9 ± 9.7	0.57
SBP (mmHg)	142.7 ± 17.2	147.6 ± 19.0	142.6 ± 16.3	0.39
DBP (mmHg)	83.8 ± 8.7	84.8 ± 9.0	82.1 ± 9.3	0.51
SO2 (%)	97 [96–97]	97 [96–97]	97 [96–97]	0.55
ACE inhibitor/ARB	16 (40.0)	9 (22.5)	15 (37.5)	0.20
Calcium blocker	4 (10.0)	2 (5.0)	7 (17.5)	0.19
Beta-blocker	5 (12.5)	6 (15.0)	3 (7.5)	0.57
Diuretic	4 (10.0)	5 (12.5)	5 (12.5)	0.92
Ezetimibe	2 (5.0)	4 (10.0)	1 (2.5)	0.35
PPI	3 (7.5)	2 (5.0)	2 (5.0)	0.86

Values are mean ± S.D. or median [25th-75th percentile] or number (percentage), *ARB* angiotensin receptor blocker, *BMI* body mass index, *DBP* diastolic blood pressure, *PASE* Physical Activity Scale for the Elderly [47], *PPI* proton pump inhibitor, *SBP* systolic blood pressure, *SO2* oxygen saturation, *VAS* Visual Analog Scale

Baseline proBNPage was inversely correlated with hemoglobin (*R* = 0.32, *P* = 0.004) and handgrip strength (*R* = 0.24, *P* = 0.03), and was directly correlated with log ESR (*R* = 0.27, *P* = 0.02). No significant correlations were found with other possible factors capable of influencing NT-proBNP variability (such as creatinine, BMI, and log CRP).

During the study, the sample was considerably reduced, not only because of group B interruption, but also due to various dropouts and cases of inadequate treatment intake. In group A in 24 months, there were eight dropouts for the following reasons: statin prescription, atrial fibrillation, lung adenocarcinoma, diffuse paresthesias, hypertension with headache and dizziness, worsening of arrhythmia, and two voluntary withdrawals. In group B in 6 months, there were 6 dropouts for the following reasons: statin prescription, atrial fibrillation, dyspepsia, two cases of diarrhea, and voluntary withdrawal. In group C, in 24 months, there were three dropouts for the following reasons: statin prescription, dyspepsia, and cerebral hemorrhage. Treatment intake was inadequate for 11 subjects in the first semester, 16 subjects in the second semester, 14 subjects in the third semester, and 17 subjects in the fourth semester.

### Effect of treatments on proBNPage

**Fig. 2:**
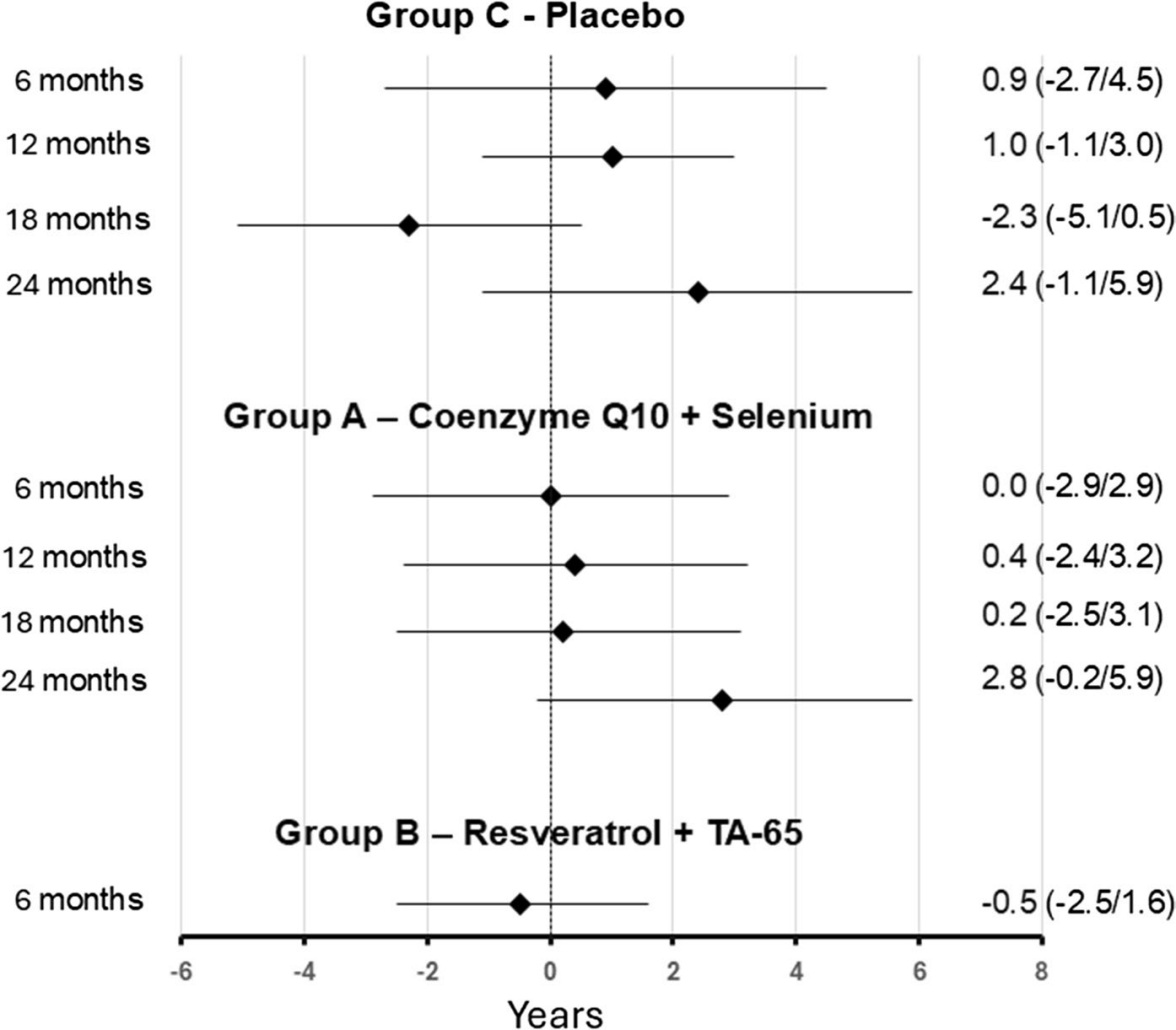
Forest plot of mean difference between proBNPage at each time and baseline, plus 95% confidence intervals, in groups C, A, and B

Per-protocol analysis – Table 2 and Fig. 2 show the changes in proBNPage from baseline to each time considered in the three groups. The baseline value was available for all subjects, but for some the value at some time later was missing (either because the subject had not shown up for the visit, or because they had not taken the treatments in adequate quantities). Thus, different sample sizes and mean baseline values are shown at each time, because for each comparison only the subjects who had both the value at that given time and the baseline value were considered. The spontaneous trend of proBNPage (group C) was much less regular than expected. After 1 year, the proBNPage had exactly increased by 1 year, and after 2 years had increased by 2.4 years, but after 6 months the increase had been 0.9 years and after 18 months there had been a drop of − 2.3 years from baseline. Due to the variability of the values and the numerical reduction of the sample, none of these variations were significant. In group A, smaller changes occurred up to 18 months, but at 24 months a sharp increase in proBNPage brought the difference from baseline to 2.8 years. None of these changes were significant in this group either. Finally, in group B, of which only measurements at baseline and at 6 months were available, there was a non-significant reduction in proBNPage of − 0.5 years. None of the differences in deltas between groups, at the same interval, were significant.

**Table 2:** ProBNPage changes in the 3 groups—per-protocol analysis

Interval	*N*	Baseline	After interval	$\Delta$	*P *value
Group C (placebo)					
0	40	68.3 ± 11.6	-	-	-
6 months	34	68.9 ± 11.6	69.8 ± 13.3	+ 0.9 ± 10.4	0.61
12 months	31	68.5 ± 12.0	69.5 ± 11.8	+ 1.0 ± 5.7	0.37
18 months	31	69.3 ± 11.8	67.0 ± 12.7	− 2.3 ± 7.7	0.10
24 months	27	71.1 ± 9.7	73.5 ± 8.5	+ 2.4 ± 8.8	0.17
Group A (Coenzyme Q10 + Selenium)					
0	40	66.3 ± 9.5	-	-	-
6 months	30	67.2 ± 9.0	67.2 ± 11.2	0 ± 7.7	1.00
12 months	26	66.9 ± 9.3	67.3 ± 9.3	+ 0.4 ± 7.0	0.76
18 months	26	66.2 ± 9.9	66.4 ± 10.5	+ 0.2 ± 6.9	0.84
24 months	25	66.6 ± 9.3	69.4 ± 9.6	+ 2.8 ± 7.5	0.07
Group B (Resveratrol + TA-65)					
0	40	65.1 ± 10.2	-	-	-
6 months	34	64.7 ± 10.1	64.2 ± 11.0	− 0.5 ± 5.8	0.64

Values are mean ± S.D. and are expressed in years, ProBNPage changes refer to the corresponding baseline values and are tested with paired *t* test, *N* and baseline values change at each interval because only subjects with both baseline and after-interval value are included in this analysis, Subjects with inadequate intake of treatments are excluded from this analysis

Intention-to-treat analysis – In this analysis, missing values of proBNPage were assigned by multiple imputation. Again, no significant changes were found within and between groups, and also the interaction term (group × time) was not significant (Supplementary Table 2). In addition, a sensitivity analysis was performed, by imputation of the highest or lowest detected values in the place of missing values, in order to promote the best or worst possible result for treatments. In the first case (best possible result, Supplementary Table 3), borderline significance could be obtained between groups and for interaction term, but without any significant progression of proBNPage over time. In the second case (worst possible result, Supplementary Table 4), in group A, a significant increase of 11.8 years with respect to baseline could be induced at the 24th month, also confirmed by a significant increase in the interaction term, but even this considerable factitious increment was not sufficient to cause a significant difference between the groups.

Linear mixed model analysis – To complete the analysis of treatment effects, we assessed a linear mixed model with proBNPage as dependent variable, including group and time as factors, and age, sex, and hemoglobin (see further) as covariates (groups A and C, all available observations: *N* = 222, Supplementary Table 5). By this analysis, in addition to age and hemoglobin, proBNPage was also significantly associated with time (24th month vs. baseline), while the associations with group and interaction term (group × time) were not significant.

### Effect of treatments on secondary outcome variables

**Fig. 3:**
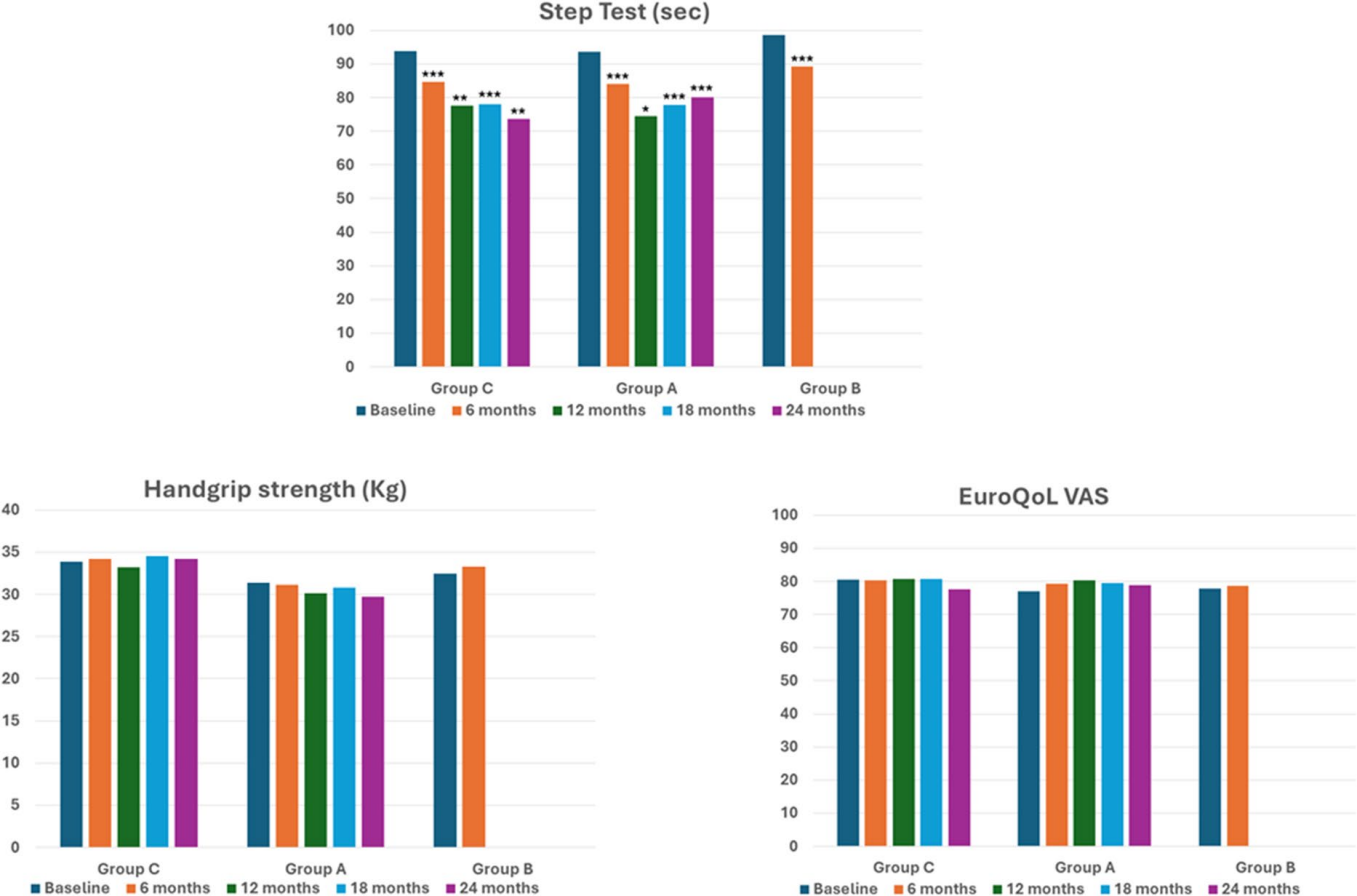
Time trends of secondary outcome variables (step test, handgrip strength, and Euro QoL VAS) in the 3 treatment groups. *P < 0.05, **P < 0.01, ***P < 0.001 vs. baseline

Figure 3 shows the trends of secondary outcome variables (step test duration, handgrip strength, and EuroQoL VAS) in the three groups during the 2 years of the study (see also Supplementary Tables 6, 7, and 8). In all groups and at all times considered, the duration of the step test was significantly reduced compared to the baseline assessment, probably due to training effect. On the other hand, no significant changes in handgrip strength and EuroQoL VAS were detected, compared to baseline, for any of the treatments and timings of the study. Linear mixed models were also applied to secondary outcome variables in groups A and C, including group and time as factors, and age and sex as covariates (Supplementary Table 5). Step test duration was associated directly with age and female sex, and inversely with time (the latter with high significance). Handgrip strength was associated with high significance with male sex and age (inverse association), and with borderline significance with group and time. EuroQoL VAS was associated with no variables. None of the secondary outcome variables were associated with group × time interaction.

### Spontaneous behavior of proBNPage in the combined group A+ C and its subgroups

The main focus of this work and the primary objective of the trial was to describe, for the first time, the progression of proBNPage in a longitudinal study, to fine tune a reliable method for assessing putative anti-aging treatments. In order to examine more precisely the spontaneous behavior of proBNPage, since no significant differences were detected between the groups A and C, we considered these two groups as a single group, including only those subjects for whom all five proBNPage values were available (Table 3). Subjects who had not taken the treatments in adequate quantities were also included in this analysis, given the apparent lack of effect of the treatments. In the sample thus composed (*N* = 68, 71.2 ± 4.4 years, 34 males and 34 females), there was a sharp increase in proBNPage at 6 months (1.4 years), followed by a progressive decrease up to − 0.7 years at 18 months compared to baseline, and then a new abrupt increase between 18 and 24 months, which brought the difference from baseline to + 2.5 years. The increase during the last semester and that between baseline and 24th month were both significant (respectively, *P* = 0.002 and *P* = 0.01). Based on the data reported in Table 3 and the correlation coefficient *R* = 0.744 between baseline and 24-month values, we can estimate that a total sample size of 260 subjects (130 per group) would be needed to obtain significant results (*P* < 0.05) within and between two groups, with a power of 80%.

**Table 3:** ProBNPage changes in groups A + C

Visit	ProBNPage (years)	$\Delta$ from baseline	*P *value
Baseline	66.8 ± 10.7	-	-
6 months	68.2 ± 12.2	+ 1.4 ± 8.9	0.20
12 months	67.6 ± 10.6	+ 0.8 ± 6.8	0.32
18 months	66.1 ± 11.5	− 0.7 ± 7.2	0.43
24 months	69.3 ± 10.7	+ 2.5 ± 7.6	0.01*

ProBNPage values are mean ± S.D, Only subjects with all 5 values are included in this analysis, which also includes those with inadequate intake of treatments (*N* = 68)

*Significant after Holm-Bonferroni correction for multiple testing

We then divided the same sample (A + C) into subgroups, each composed of 34 subjects (males vs. females, “younger” vs. “older,” and low vs. high baseline proBNPage), to ascertain whether the change observed at 24 months concerned any subgroup in particular (Table 4). The subjects with chronological age ≤ the median (70 years) were considered “young,” and a proBNPage ≤ the median (68 years) was considered low. The largest changes after 2 years occurred in the subgroup of the older subjects (+ 3.0 years, *P* = 0.009) and in the subgroup with low baseline proBNPage (+ 4.0 years, *P* = 0.006), and both changes retained their significance after Holm-Bonferroni correction for multiple testing. The smallest change after 2 years was in subjects with high baseline proBNPage: only + 0.8 years (*P* = 0.50). By ANOVA for repeated measures, the low baseline proBNPage factor was associated with significant within (*P* = 0.003) and between (*P* < 0.0001) effects. Similarly, the “older” factor was associated with significant within (*P* = 0.01) and between (*P* < 0.05) effects. However, probably due to the small size of the subgroups, both interactions (time × low baseline proBNPage and time × “older”) were not significant.

**Table 4:** Subgroup analysis: proBNPage changes in groups A + C

Subgroup	*N*	Baseline	After 24 months	$\Delta$	*P *value
Males	34	65.8 ± 9.2	67.9 ± 10.4	+ 2.1 ± 5.8	0.045
Females	34	67.9 ± 12.1	70.6 ± 10.9	+ 2.7 ± 9.2	0.09
Younger	34	64.8 ± 12.9	66.6 ± 11.1	+ 1.8 ± 8.8	0.24
Older	34	68.9 ± 7.5	71.9 ± 9.7	+ 3.0 ± 6.3	0.009*
Low baseline proBNPage	34	58.6 ± 7.9	62.6 ± 9.4	+ 4.0 ± 8.1	0.006*
High baseline proBNPage	34	75.1 ± 5.4	75.9 ± 7.2	+ 0.8 ± 6.9	0.50

Values are mean ± S.D. and are expressed in years, Only subjects with all 5 values are included in this analysis, which also includes those with inadequate intake of treatments, Younger = Baseline chronological age ≤ median value (70 years) in groups A + C, Low baseline proBNPage = Baseline proBNPage ≤ median value (66 years) in groups A + C

*Significant after Holm-Bonferroni correction for multiple testing

By enrolling subjects over 70 years of age, based on the data reported in Table 4 and the correlation coefficient *R* = 0.759 between baseline and 24-month values, we can estimate that a total sample size of 126 subjects (63 per group) would be sufficient to obtain significant results (*P* < 0.05) within and between two groups, with a power of 80%.

### Analysis of proBNPage variability

To better understand the possible causes of proBNPage variability in our sample, in a linear mixed model with proBNPage as dependent variable, we included sex, baseline age, current (at times 0, 12, and 24 months) age, hemoglobin, log ESR, log INR, creatinine, log CRP, and BMI as covariates (groups A + C together, *N* = 80, number of observations = 222). After a backward elimination procedure, only current age (*P* < 0.0001) and hemoglobin (*P* = 0.0009) remained in the model, with the following coefficients: proBNPage = current age × 0.61 (95% CI 0.32/0.89) − current hemoglobin × 3.19 (95% CI − 4.96/− 1.42) + 68.30 (95% CI 66.97/69.63). Thus, among the safety variables measured at times 0, 12, and 24 months, only hemoglobin was found to be a significant factor of variability for proBNPage.

### Safety analysis

**Fig. 4:**
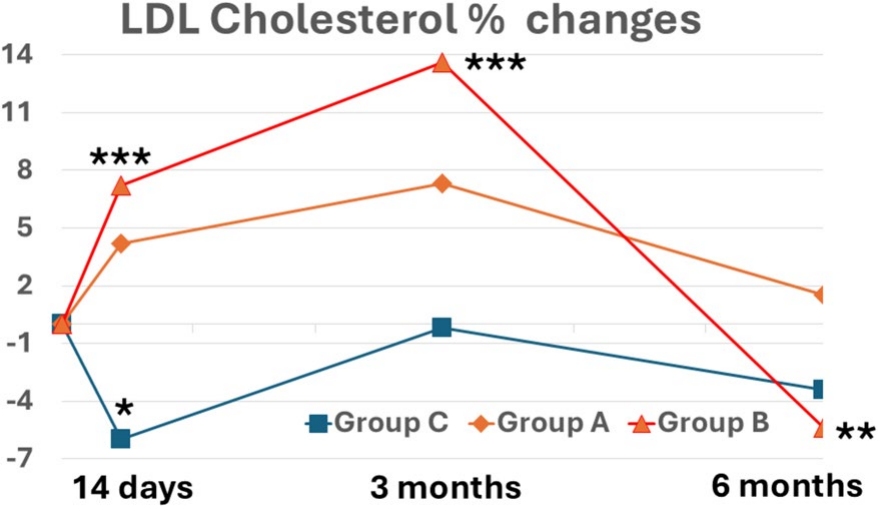
Time trends of LDL cholesterol percent changes in the 3 treatment groups during the first 6 months of study. At the 5th month treatment intake was discontinued in group B. *P < 0.05, **P < 0.01, ***P < 0.001 vs. baseline

Table 5 summarizes the main clinical and laboratory data on treatment safety. At month 24, there was a significant drop in INR and an increase in ESR in both groups A and C (perhaps favored by the spread of flu syndromes at that time). Similarly, in both groups A and C, there was a slight increase in blood glucose, which was instead associated with a significant decrease in glycated hemoglobin. In addition, an increase in HDL cholesterol was detected in all groups. The most significant changes occurred in group B, resulting in the discontinuation of the study in this group. In fact, as early as 14 days, very significant increases in total cholesterol, LDL cholesterol, triglycerides, potassium, calcium, and ALT were detected. The most marked change concerned LDL cholesterol (+ 13.6% at 3 months, *P* < 0.0001, Fig. 4, Supplementary Tables 9 and 10), but after the discontinuation of treatments, already at the 6th month LDL cholesterol had dropped to − 5.4% compared to baseline (*P* = 0.002).

**Table 5:** Safety analysis: clinical and laboratory variables

Variable	Group C		Group A		Group B	
	(Placebo, *N* = 37)		(Coenzyme Q10 + Selenium, *N* = 25)		(Resveratrol + TA-65, *N* = 34)	
	Baseline	24 months	Baseline	24 months	Baseline	3 months
BMI (kg/m^2^)	26.4 ± 4.1	26.6 ± 3.9	25.3 ± 4.0	25.5 ± 3.7	26.6 ± 4.7	26.8 ± 4.8**
SBP (mmHg)	141.2 ± 16.6	137.1 ± 14.9	144.8 ± 18.6	140.8 ± 23.3	143.5 ± 15.9	142.9 ± 14.9
DBP (mmHg)	83.9 ± 8.1	82.3 ± 7.5	83.6 ± 10.3	82.5 ± 11.2	82.0 ± 9.2	83.0 ± 11.8
Heart rate (beats/min)	69.5 ± 11.6	66.4 ± 10.1	63.7 ± 8.7	63.1 ± 9.7	67.3 ± 7.9	65.6 ± 9.0
QTc (ms)	421.6 ± 22.6	425.2 ± 22.2	426.2 ± 22.1	420.2 ± 19.6	424.2 ± 14.9	415.8 ± 16.9**
Leukocytes (10^9^/L)	6.11 ± 1.00	6.01 ± 1.21	5.83 ± 1.09	5.77 ± 1.18	5.92 ± 1.01	6.24 ± 1.55
Neutrophils (%)	59.3 ± 8.4	61.2 ± 8.7*	57.0 ± 8.8	57.5 ± 8.0	59.1 ± 7.3	59.6 ± 8.0
Lymphocytes (%)	30.8 ± 7.9	29.0 ± 7.7*	32.8 ± 8.2	32.5 ± 7.1	31.0 ± 6.9	30.0 ± 7.5
Eosinophils (%)	2.6 [1.4–3.4]	2.7 [1.8–3.7]	2.2 [1.4–3.2]	2.4 [1.7–3.1]	2.1 [1.5–2.4]	2.4 [1.6–3.8]
Hemoglobin (g/dL)	14.3 ± 1.5	14.3 ± 1.1	14.0 ± 1.1	14.2 ± 0.9	14.5 ± 1.2	14.6 ± 1.2
Platelets (10^9^/L)	227.4 ± 51.6	240.0 ± 54.5*	247.8 ± 51.5	243.4 ± 58.4	232.8 ± 51.7	234.4 ± 49.7
ESR (mm/h)	8.0 [2.0–12.0]	**13.0 [6.0**–22.0]***	5.0 [3.0–12.0]	**9.0 [7.0**–18.0]***	9.0 [4.3–15.0]	8.5 [5.0–14.0]
INR	1.02 [1.00–1.06]	**0.92 [0.89**–0.95]***	1.05 [1.03–1.08]	**0.92 [0.91**–0.95]***	1.04 [1.01–1.06]	1.02 [0.97–1.06]*
Creatinine (mg/dL)	0.85 ± 0.16	0.82 ± 0.15	0.84 ± 0.16	0.84 ± 0.18	0.90 ± 0.15	0.86 ± 0.11*
Blood Glucose (mg/dL)	83 [77–91]	86.0 [80.0–95.0]*	82.0 [77.0–89.0]	89.0 [84.0–94.0]*	83.0 [75.5–87.0]	88.5 [80.3–94. 8]**
HbA1c (mmol/mol)	36.3 ± 3.5	35.1 ± 3.6***	36.2 ± 3.1	34.9 ± 3.9***	36.7 ± 3.1	36.8 ± 2.8
Cholesterol (mg/dL)	213.1 ± 27.5	211.3 ± 34.7	211.7 ± 23.5	217.8 ± 27.7	213.2 ± 30.1	238.5 ± 38.9***
LDL cholesterol (mg/dL)	132.1 ± 21.5	126.4 ± 28.9	126.7 ± 18.0	130.7 ± 21.6	130.6 ± 23.0	148.0 ± 29.1***
HDL cholesterol (mg/dL)	60.7 ± 13.9	64.2 ± 14.9**	67.2 ± 10.4	70.3 ± 13.7*	62.1 ± 14.9	65.8 ± 14.2**
Triglycerides (mg/dL)	89.0 [67.0–117.0]	96.0 [85.0–103.0]	84.0 [68.0–95.0]	84.0 [70.0–97.0]	88.0 [66.0–127.8]	101.0 [79.5–150.8]***
Sodium (mmol/L)	141.7 ± 1.9	140.0 ± 2.4***	141.2 ± 1.9	140.0 ± 1.4	141.6 ± 1.8	140.9 ± 2.3*
Potassium (mmol/L)	4.2 ± 0.4	4.2 ± 0.3	4.4 ± 0.3	4.2 ± 0.3*	4.3 ± 0.3	4.6 ± 0.4**
Calcium (mg/dL)	9.4 ± 0.4	9.4 ± 0.3	9.4 ± 0.4	9.4 ± 0.3	9.5 ± 0.4	9.7 ± 0.3**
AST ((U/L)	23.0 [20.0–26.0]	22.0 [21.0–27.0]	23.0 [20.0–25.0]	23.0 [21.0–24.0]	22.0 [19.0–25.0]	23.0 [21.0–26.8]*
ALT (U/L)	18.0 [15.0–22.0]	17.0 [14.0–24.0]	16.0 [13.0–21.0]	15.0 [14.0–21.0]	15.0 [13.0–20.0]	19.0 [16.3–24.3]**
GammaGT (U/L)	25.0 [17.0–37.0]	20.0 [16.0–31.0]	23.0 [17.0–29.0]	20.0 [16.0–26.0]	22.0 [18.0–28.3]	22.5 [18.0–26.0]
CRP (mg/dL)	0.17 [0.08–0.35]	0.22 [0.13–0.35]	0.10 [0.08–0.13]	0.11 [0.08–0.24]	0.22 [0.10–0.45]	0.20 [0.16–0.34]
PSA (ng/mL)	1.41 [0.98–2.92] (*N* = 17)	1.63 [1.27–2.11] (*N* = 17)	0.93 [0.61–1.43] (*N* = 11)	0.73 [0.58–1.42] (*N* = 11)	0.95 [0.52–1.36] (*N* = 15)	0.73 [0.40–1.48] (*N* = 15)

Values are mean ± S.D. or median [25th-75th percentile], *ALT* alanine aminotransferase, *AST* aspartate aminotransferase, *BMI* body mass index, *CRP* C-reactive protein, *DBP* diastolic blood pressure, *ESR* erythrocyte sedimentation rate, *GammaGT* gamma glutamyl transferase, *HDL* high density lipoprotein, *INR* international normalized ratio, *LDL* low density lipoprotein, *PSA* prostatic specific antigen, *QTc* corrected QT interval, *SBP* systolic blood pressure

*P < 0.05; **P < 0.01; ***P < 0.001. *P* values refer to the corresponding baseline values and were obtained with paired *t* test or Wilcoxon’s test, as appropriate. Only subjects with adequate intake of treatments were included in the analysis of groups A and B. In bold significant changes after Holm-Bonferroni correction for multiple testing

Table 6 shows the number and incidence of the adverse events that occurred during the study (mostly during the first 6 months). In group B, gastrointestinal disorders occurred in greater numbers than in the other groups: abdominal pain, aerophagy, flatulence, and especially diarrhea (5 per 100 person-months vs. 0.21 in each of the other two groups, *P* < 0.0001). Although in most cases these events were graded by participants as mild, in seven cases they were graded as severe and were a cause of withdrawal from the study.

**Table 6:** Adverse events

Adverse event	Group C	Group A	Group B
Diarrhea	2 (0.21)	2 (0.21)	12 (5.00)***
Abdominal pain	2 (0.21)	0	5 (2.08)**
Aerophagy	0	0	2 (0.83)
Flatulence	0	0	2 (0.83)
Nausea	1 (0.10)	1 (0.10)	2 (0.83)
Increased appetite	2 (0.21)	1 (0.10)	0
Xerostomia	1 (0.10)	2 (0.21)	2 (0.83)
Constipation	2 (0.21)	3 (0.31)	0
Nycturia	1 (0.10)	0	1 (0.42)
Pollakiuria	1 (0.10)	0	1 (0.42)
Dark urine	0	0	3 (1.25)
Insomnia	1 (0.10)	3 (0.31)	2 (0.83)
Somnolence	1 (0.10)	2 (0.21)	2 (0.83)
Headache	0	2 (0.21)	2 (0.83)
Asthenia	2 (0.21)	0	1 (0.42)
Itching	3 (0.31)	0	1 (0.42)
Myalgia	1 (0.10)	0	2 (0.83)
Other	8 (0.83)	8 (0.83)	6 (2.50)*

Values are count and incidence per 100 person-months

***P < 0.0001; **P < 0.01; *P < 0.05 (comparisons vs. group C)

## Discussion

With this pilot study, we have described the longitudinal course of proBNPage, and have understood how future studies aimed at using proBNPage to evaluate the effect of anti-aging treatments should be designed. In addition to this primary objective, the trial had the secondary objective of testing two pairs of possible anti-aging treatments. None of the treatments examined induced significant changes in proBNPage and secondary outcome variables, but the small sample size, caused by many dropouts and non-optimal treatment intake, does not allow us to exclude possible effects of the treatments. Finally, this study showed a marked cholesterol increase in group B, which led to the discontinuation of the trial at the 5th month in this group.

### ProBNPage as a method to evaluate the effectiveness of anti-aging treatments

In our sample, mean proBNPage was lower than chronological age. This finding is probably related to the fact that the method of calculating proBNPage had previously been obtained in an unselected sample (apart from the exclusion of cases of heart failure and atrial fibrillation) [7], while the current sample included people who were as healthy as possible (and therefore on average “younger” than their chronological age).

Based on data from the literature and our previous experiences in cross-sectional studies, which had demonstrated the progression of NT-proBNP and proBNPage in groups of subjects with increasing age [7, 8], we hypothesized that such behavior could also occur in subjects studied longitudinally. In particular, we thought that the increment of proBNPage from baseline would progressively increase over time, until it became significant using statistical tests for paired data. Actually, our previous cross-sectional data had shown that, over a period of 1 year, decreases, rather than increases, in proBNPage could often occur, while over a period of 2 years the finding of an increase in proBNPage was almost constant [12]. For this reason, we chose a duration of 2 years for our trial, also thinking that a 2-year increase could be the minimal clinically important difference for proBNPage. The present study has shown that the longitudinal trend of proBNPage is also rather irregular, with increases followed by decreases and vice versa, confirming that only after 2 years a significant increment from baseline is obtained.

The main known causes of NT-proBNP variability, in addition to heart disease, sex, and age, are renal failure [39], anemia [40], and obesity [41]. It is also possible that inflammatory conditions and strenuous physical activity may promote transient increases, followed by subsequent decreases, in NT-proBNP [42–45]. In our sample, after multivariate analysis, we could only confirm an inverse independent relationship between proBNPage and hemoglobin: a decrease by 1 g/dl in hemoglobin was associated with an increase by 3.19 years in proBNPage.

This study showed that the subgroup with the greatest propensity to exhibit an increase in proBNPage at 2 years was the one that initially had proBNPage values below the median, while the subgroup with higher values did not achieve a significant increase. This fact is at least partly due to the mechanism of regression towards the mean [46], which is often observed in longitudinal studies. In our case, many individuals with proBNPage values above the median likely had factors, such as those mentioned above, that, regardless of age, cause an increase in NT-proBNP concentration. In these subjects, the progression of proBNPage with age is less visible, and even reductions may occur because of the normalization of confounding factors. Conversely, in subjects with proBNPage below the median, confounding factors were not present, or acted in the opposite direction, so that the effect of age was more evident or amplified. Even at the individual level, these mechanisms could favor increases and subsequent decreases in values, causing proBNPage to take on an apparently capricious trend when evaluated over relatively short time intervals and in small groups.

To facilitate the use of proBNPage in future studies, it may be useful to take advantage of the fact that its progression after 2 years was more evident and significant in older subjects (over 70) than in younger subjects (+ 3.0 years, *P* = 0.009 vs. + 1.8 years, *P* = 0.24, respectively). The relationship of NT-proBNP with age is exponential, so its concentration is proportionally higher in older than in younger subjects. For example, in men, at the age of 65, an age increment of 1 year is associated with an increase in NT-proBNP of 5 pg/ml, while at the age of 75 the same increment is associated with an increase in NT-proBNP of 13 pg/ml. It is thus possible that in older subjects the age factor may influence NT-proBNP levels to a greater extent than confounding factors. We therefore propose to increase the age of entry into the sample from 65 to 70 years. Using the statistical parameters obtained in the subgroup aged > 70 years, we calculated that, in a 2-year study, for the comparison between two groups (placebo vs. treatment) a minimum sample size of 63 subjects per group could allow the demonstration of significant changes within, and also between, the groups, with a power of 80%. Admitting dropout rates of up to 30%, 90 subjects per group should be enrolled (180 for two groups). This objective could be easily achieved by replicating our convocation mechanism, i.e., 2000 invitation letters with questionnaire to pre-selected subjects.

### Coenzyme Q10 + selenium association

In group A, compared to placebo, proBNPage had an apparently slower progression in the first year and an apparently faster progression in the second year, but none of these changes were significant. Similarly, none of the secondary outcome variables behaved differently between this group and the placebo group. However, we cannot exclude that in a larger sample or with a longer observation period, different results may be obtained. In fact, this association had previously favored a reduction in mortality and NT-proBNP levels in a larger sample than ours after 4 years of treatment [21], and these results were maintained even after 12 years [22]. In addition, a double dose of selenium was used, compared to the 100 mcg/day allowed in Italy.

### Resveratrol + TA*-*65 association and hypercholesterolemic effect

Unfortunately, the intake of treatments in group B was interrupted at the 5th month, so we only have data for the first semester of the study. Although proBNPage at baseline was the lowest of the three groups, at the 6th month, there was an apparent decrease of 0.5 years in proBNPage, which however was not significant. Many participants presented gastrointestinal disorders and a significant increase in cholesterolemia. There are several literature data suggesting that the component mainly responsible for these adverse events might be high-dose resveratrol [32], while previously there had never been any report of similar disorders caused by TA-65. In particular, in September 2022, the meta-analysis by Cao et al. [33] showed that the known cholesterol-lowering action of resveratrol had been obtained only in studies that had used daily doses below 500 mg, while for higher doses the effect was reversed, becoming hypercholesterolemic. This is consistent with our study, in which the dose of 700 mg/day was used. This result suggests that resveratrol may act more like a drug than like a dietary supplement, causing adverse effects in the event of an overdose, while possible beneficial effects might occur at lower doses. Further studies are warranted to separately evaluate the effect on biological age of TA-65 and resveratrol at doses below 500 mg/day.

The increase in HDL cholesterol is not attributable to the same mechanisms, having also occurred in groups A and C. A possible explanation could be an incentive to increase physical activity induced by participation in the trial, as the generalized improvement in performance at the step test also seems to suggest.

### Limitations

Using for the first time parameters obtained from a longitudinal study on proBNPage, we have shown that the sample used in this study, whose size was based on a previous cross-sectional study, was underpowered. This limitation was further compounded by dropouts and inadequate intake of the treatments, which prevented us from drawing definitive conclusions on the effects of the treatments and repositions these findings as exploratory.

This study did not compare proBNPage to other aging biomarkers, as the method required further development. Now that essential longitudinal data is available, future studies may compare this system with existing ones and perhaps it will be possible to know whether proBNPage reflects the same, complementary, or entirely different aspects of biological aging.

Although the method to follow proBNPage progression over time is now better understood, we still do not know whether proBNPage can be affected by any treatment, and whether reducing NT-proBNP concentrations and proBNPage (a surrogate of biological age) would lead to real benefits on aging and aging-related diseases. However, the latter limitation also applies to all other methods of biological age estimation.

## Conclusions

Despite the irregular fluctuations of proBNPage, this pilot study was able to achieve its primary objective, i.e., the development of a method for assessing a surrogate of biological age, based on serial measurements of NT-proBNP, to be used in subsequent studies aimed at evaluating the effect of potential anti-aging treatments. In particular, we have calculated the sample size based on longitudinal data, have confirmed that a trial duration of 2 years is needed to obtain significant results, and have found that more significant results might be obtained enrolling older subjects. No significant benefits could be demonstrated for any of the treatments tested, but this does not exclude their possible effects, in view of the low power of our study, compounded by the high dropout rate. Finally, the marked increase in cholesterolemia obtained in group B suggests that the administration of resveratrol in high doses should be avoided.

## Supplementary Information

Below is the link to the electronic supplementary material.

ESM 1 (DOCX 46.4 KB)

## Data Availability

All data collected during this study will be provided in pseudonym form upon reasonable request.
