# Supplementary material for: The proBNPage reduction (PBAR) trial—results of a randomized, double blind, placebo-controlled, pilot study to fine tune an NT-proBNP-based method to assess the effect of anti-aging treatments
Source: GeroScience. 2025 Sep 8;48(3):3939–55. doi: 10.1007/s11357-025-01827-y (PMC13355970; doi:10.1007/s11357-025-01827-y)
Supplement: Supplementary file 1 — (DOCX 46.4 KB) [file 11357_2025_1827_MOESM1_ESM.docx]

**Supplementary Table 1 –** Inclusion and exclusion criteria

| *Inclusion criteria* | *Exclusion criteria* |
| --- | --- |
|  |  |
| 1.Age ranging between 65 and 80 years | 1.History of heart failure |
| 2.Signed informed consent | 2.Previous or current atrial fibrillation |
|  | 3.History of coronary artery disease (myocardial infarction or angina pectoris) |
|  | 4.Taking any of the dietary supplements of the present study during the 2 previous months |
|  | 5.Taking a statin or monacolin K or dietary supplements with red yeast rice |
|  | 6.Taking anticoagulant drugs |
|  | 7.Taking more than 4 drugs a day |
|  | 8.Hospital admission during the last 3 years |
|  | 9.Known allergy to any food supplement of the present study |
|  | 10.Malignant neoplasm in progress |
|  | 11.Any other severe disease which, in the opinion of the investigator, contraindicates the participation in the study |
|  | 12.Alcohol or drug abuse |
|  | 13.Participation in other trial |
|  | 14.Uncertainty of the subject about his/her willingness to follow the study for 2 years |
|  | 15.Deficit of one or more Activities of Daily Living |
|  | 16.Relevant alterations in the ECG or in laboratory results which, in the opinion of the investigator, contraindicate participation in the study |

**Supplementary Table 2** – ProBNPage changes in the 3 groups Intention-to-treat analysis

| *Visit* | *ProBNPage (years)* | *Δ from baseline* | *P value* |
| --- | --- | --- | --- |
| **Group C (Placebo, N=40)** | | | |
| Baseline | 68.3 ± 11.6 | - | - |
| 6 months | 69.4 ± 13.8 | + 1.1 ± 11.4 | 0.54 |
| 12 months | 68.9 ± 12.1 | + 0.6 ± 7.1 | 0.55 |
| 18 months | 66.4 ± 12.1 | - 1.9 ± 7.2 | 0.12 |
| 24 months | 69.9 ± 11.2 | + 1.6 ± 7.8 | 0.20 |
|  |  |  |  |
| **Group A (Coenzyme Q10 + Selenium, N=40)** | | | |
| Baseline | 66.3 ± 9.5 | - | - |
| 6 months | 66.7 ± 11.5 | + 0.4 ± 8.4 | 0.75 |
| 12 months | 66.2 ± 9.9 | - 0.1 ± 7.5 | 0.93 |
| 18 months | 66.3 ± 10.6 | 0 ± 6.7 | 0.98 |
| 24 months | 69.1 ± 9.4 | + 2.8 ± 7.6 | 0.02* |
|  |  |  |  |
| **Group B (Resveratrol + TA-65, N=40)** | | | |
| Baseline | 65.1 ± 10.2 | - | - |
| 6 months | 63.0 ± 11.1 | - 2.1 ± 7.5 | 0.08 |

Intention-to-treat analysis with assignment of missing values by multiple imputation.

ProBNPage values are mean ± S.D.

ANOVA for repeated measures: Within group P value = 0.02, Between group P value = 0.46, Interaction (group x time) P value = 0.55.

*Nonsignificant after Holm-Bonferroni correction for multiple testing.

**Supplemetary Table 3** – ProBNPage changes in the 3 groups Sensitivity analysis: best case for treatments

| *Visit* | *ProBNPage (years)* | *Δ from baseline* | *P value* |
| --- | --- | --- | --- |
| **Group C (Placebo, N=40)** | | | |
| Baseline | 68.3 ± 11.6 | - | - |
| 6 months | 71.5 ± 16.4 | + 3.2 ± 13.5 | 0.14 |
| 12 months | 71.0 ± 15.3 | + 2.7 ± 10.7 | 0.11 |
| 18 months | 69.8 ± 17.5 | + 1.5 ± 13.5 | 0.49 |
| 24 months | 73.1 ± 16.2 | + 4.8 ± 12.9 | 0.02* |
|  |  |  |  |
| **Group A (Coenzyme Q10 + Selenium, N=40)** | | | |
| Baseline | 66.3 ± 9.5 | - | - |
| 6 months | 64.9 ± 13.8 | - 1.4 ± 13.0 | 0.49 |
| 12 months | 62.3 ± 14.3 | - 4.0 ± 14.3 | 0.08 |
| 18 months | 61.2 ± 15.5 | - 5.1 ± 15.2 | 0.04* |
| 24 months | 61.7 ± 17.4 | - 4.6 ± 17.6 | 0.10 |
|  |  |  |  |
| **Group B (Resveratrol + TA-65, N=40)** | | | |
| Baseline | 65.1 ± 10.2 | - | - |
| 6 months | 59.4 ± 15.4 | - 5.7 ± 14.2 | 0.02* |

Intention-to-treat analysis with imputation for missing values with maximum detected value (114 years) in group C, and minimum detected value (32 years) in groups A and B.

ProBNPage values are mean ± S.D.

ANOVA for repeated measures: Within group P value = 0.37, Between group P value = 0.01, Interaction (group x time) P value = 0.01.

*Nonsignificant after Holm-Bonferroni correction for multiple testing.

**Supplemetary Table 4** – ProBNPage changes in the 3 groups Sensitivity analysis: worst case for treatments

| *Visit* | *ProBNPage (years)* | *Δ from baseline* | *P value* |
| --- | --- | --- | --- |
| **Group C (Placebo, N=40)** | | | |
| Baseline | 68.3 ± 11.6 | - | - |
| 6 months | 67.4 ± 15.4 | - 0.9 ± 13.4 | 0.69 |
| 12 months | 66.9 ± 14.2 | - 1.4 ± 10.4 | 0.43 |
| 18 months | 63.6 ± 15.1 | - 4.7 ± 12.6 | 0.02* |
| 24 months | 67.0 ± 15.0 | - 1.3 ± 14.0 | 0.57 |
|  |  |  |  |
| **Group A (Coenzyme Q10 + Selenium, N=40)** | | | |
| Baseline | 66.3 ± 9.5 | - | - |
| 6 months | 71.0 ± 16.0 | + 4.7 ± 15.8 | 0.07 |
| 12 months | 72.5 ± 18.0 | + 6.2 ± 18.0 | 0.04* |
| 18 months | 73.5 ± 19.5 | + 7.2 ± 18.5 | 0.02* |
| 24 months | 78.1 ± 20.2 | + 11.8 ± 19.3 | 0.0004 |
|  |  |  |  |
| **Group B (Resveratrol + TA-65, N=40)** | | | |
| Baseline | 65.1 ± 10.2 | - | - |
| 6 months | 71.7 ± 20.7 | + 6.6 ± 18.4 | 0.03* |

Intention-to-treat analysis with imputation for missing values with minimum detected value (32 years) in group C, and maximum detected value (114 years) in groups A and B.

ProBNPage values are mean ± S.D.

ANOVA for repeated measures: Within-group P value = 0.01, Between-group P value = 0.06, Interaction (group x time) P value = 0.0002.

*Nonsignificant after Holm-Bonferroni correction for multiple

testing.

**Supplementary Table 5** – Linear mixed models for primary and secondary variables

|  | proBNPage  (years) | Step test duration  (sec) | Handgrip strength  (Kg) | EuroQoL VAS  (0-100) |
| --- | --- | --- | --- | --- |
|  |  |  |  |  |
| Number of observations | 222 | 364 | 366 | 367 |
| Fixed effects Omnibus test (P value) |  |  |  |  |
| Group | 0.40 | 0.61 | 0.03 | 0.45 |
| Time | 0.008 | <0.0001 | 0.03 | 0.58 |
| Male sex | 0.56 | 0.02 | <0.0001 | 0.65 |
| Age | 0.008 | 0.003 | 0.0006 | 0.45 |
| Hemoglobin | 0.0002 | - | - | - |
| Group x Time | 0.73 | 0.77 | 0.69 | 0.44 |
| Coefficients (Estimate, 95% C.I.) |  |  |  |  |
| Group A vs. C | -1.714, -5.683/2.255 | 2.117, -6.000/10.233 | -2.626, -4.977/-0.275 | -1.594, -5.682/2.493 |
| Time 6 mo. vs. 0 | - | -9.015, -13.411/-4.618 | 0.286, -0.322/0.894 | 0.904, -0.967/2.775 |
| Time 12 mo. vs. 0 | 0.53, -1.139/2.199 | -16.762, -21.245/-12.278 | -0.232, -0.852/0.388 | 1.358, -0.525/3.241 |
| Time 18 mo. vs. 0 | - | -15.301, -19.824/-10.779 | 0.574, -0.046/1.194 | 0.972, -0.928/2.871 |
| Time 24 mo. vs. 0 | 2.606, 0.897/4.314 | -17.181, -21.706/-12.655 | -0.326, -0.953/0.300 | 0.150, -1.769/2.068 |
| Male sex | -1.246, -5.393/2.901 | -9.763, -17.969/-1.558 | 16,449, 14.064/18,834 | 0.960, -3.177/5.097 |
| Age | 0.605, 0.170/1.040 | 1.418, 0.525/2.311 | -0.472, -0.729/-0.214 | -0.173, -0.622/0.276 |
| Hemoglobin | -2.307, -3.526/-1.089 | - | - | - |
| Intercept | 68.215, 66.238/70.192 | 83.411, 79.364/87.458 | 33.002, 31.828/34.175 | 79.577, 77.538/81.615 |

Data of groups A and C are included in the analysis. Hemoglobin, a significant source of proBNPage variability, was not available at times 6 and 18 months.

**Supplementary Table 6** – Step test changes in the 3 groups

| *Interval* | *N* | *Baseline* | *After interval* | *P value* |
| --- | --- | --- | --- | --- |
| **Group C (Placebo)** | | | | |
| 0 | 40 | 94.6 ± 25.3 | - | - |
| 6 months | 34 | 92.4 ± 24.3 | 84.7 ± 19.7 | <0.0001 |
| 12 months | 31 | 94.2 ± 24.2 | 77.6 ± 22.4 | 0.007 |
| 18 months | 30 | 91.1 ± 18.0 | 78.1 ± 14.1 | <0.0001 |
| 24 months | 27 | 94.5 ± 25.1 | 73.6 ± 21.6 | 0.003 |
| **Group A (Coenzyme Q10 + Selenium)** | | | | |
| 0 | 40 | 95.5 ± 24.6 | - | - |
| 6 months | 30 | 90.9 ± 17.8 | 84.0 ± 23.2 | 0.0008 |
| 12 months | 24 | 91.3 ± 19.7 | 74.6 ± 26.1 | 0.01 |
| 18 months | 25 | 89.5 ± 16.2 | 77.9 ± 17.9 | <0.0001 |
| 24 months | 25 | 91.6 ± 19.3 | 80.2 ± 22.6 | <0.0001 |
| **Group B (Resveratrol + TA-65)** | | | | |
| 0 | 40 | 97.3 ± 19.3 | - | - |
| 6 months | 34 | 98.7 ± 19.8 | 89.2 ± 17.8 | <0.0001 |

Values are mean ± S.D. Step test results are expressed in sec.

Step test changes refer to the corresponding baseline values and are tested with paired t test. All changes are significant after Holm-Bonferroni correction for multiple testing.

N and baseline values change at each interval because only subjects with both baseline and after-interval value are included in the analysis.

Subjects with inadequate intake of treatments are excluded from this analysis.

**Supplementary Table 7** – Handgrip strength changes in the 3 groups

| *Interval* | *N* | *Baseline* | *After interval* | *P value* |
| --- | --- | --- | --- | --- |
| **Group C (Placebo)** | | | | |
| 0 | 40 | 34.1 ± 10.2 | - | - |
| 6 months | 34 | 33.7 ± 10.5 | 34.2 ± 10.7 | 0.30 |
| 12 months | 31 | 33.0 ± 9.5 | 33.2 ± 10.3 | 0.70 |
| 18 months | 31 | 33.3 ± 10.1 | 34.5 ± 10.7 | 0.054 |
| 24 months | 27 | 34.0 ± 10.7 | 34.2 ± 10.6 | 0.72 |
| **Group A (Coenzyme Q10 + Selenium)** | | | | |
| 0 | 40 | 31.8 ± 8.9 | - | - |
| 6 months | 30 | 30.7 ± 8.4 | 31.1 ± 8.6 | 0.28 |
| 12 months | 24 | 30.3 ± 8.5 | 30.1 ± 9.7 | 0.78 |
| 18 months | 26 | 30.5 ± 8.2 | 30.8 ± 9.2 | 0.53 |
| 24 months | 25 | 30.0 ± 8.5 | 29.7 ± 9.1 | 0.54 |
| **Group B (Resveratrol + TA-65)** | | | | |
| 0 | 40 | 33.1 ± 11.2 | - | - |
| 6 months | 34 | 32.5 ± 10.6 | 33.3 ± 11.0 | 0.08 |

Values are mean ± S.D. Step test results are expressed in Kg.

Handgrip strength changes refer to the corresponding baseline values and are tested with paired t test.

N and baseline values change at each interval because only subjects with both baseline and after-interval value are included in the analysis.

Subjects with inadequate intake of treatments are excluded from this analysis.

**Supplementary Table 8** – EuroQoL 5D visual analog scale changes in the 3 groups

| *Interval* | *N* | *Baseline* | *After interval* | *P value* |
| --- | --- | --- | --- | --- |
| **Group C (Placebo)** | | | | |
| 0 | 40 | 80.5 ± 10.2 | - | - |
| 6 months | 33 | 80.7 ± 10.9 | 80.3 ± 9.8 | 0.79 |
| 12 months | 31 | 81.1 ± 10.5 | 80.7 ± 10.0 | 0.79 |
| 18 months | 31 | 80.3 ± 11.0 | 80.7 ± 9.7 | 0.82 |
| 24 months | 27 | 79.6 ± 11.2 | 77.7 ± 12.3 | 0.22 |
| **Group A (Coenzyme Q10 + Selenium)** | | | | |
| 0 | 40 | 77.4 ± 11.2 | - | - |
| 6 months | 30 | 79.0 ± 9.1 | 79.2 ± 7.6 | 0.88 |
| 12 months | 26 | 78.6 ± 9.5 | 80.4 ± 9.3 | 0.23 |
| 18 months | 26 | 79.2 ± 9.7 | 79.4 ± 13.1 | 0.92 |
| 24 months | 25 | 78.8 ± 9.6 | 78.8 ±11.0 | 0.98 |
| **Group B (Resveratrol + TA-65)** | | | | |
| 0 | 40 | 78.3 ±8.9 | - | - |
| 6 months | 34 | 77.9 ± 8.9 | 78.7 ± 10.5 | 0.59 |

Values are mean ± S.D. EuroQoL 5D VAS results are expressed on a 0-100 scale.

EuroQoL 5D VAS changes refer to the corresponding baseline values and are tested with paired t test.

N and baseline values change at each interval because only subjects with both baseline and after-interval value are included in the analysis.

Subjects with inadequate intake of treatments are excluded from this analysis.

**Supplementary Table 9** – Early LDL cholesterol changes in the 3 groups

| *Interval* | *N* | *Baseline* | *After interval* | *% Δ* | *P value* |
| --- | --- | --- | --- | --- | --- |
| **Group C (Placebo)** | | | | | |
| 0 | 39 | 137.2 ± 20.7 | - | - | - |
| 14 days | 38 | 132.7 ± 21.0 | 124.6 ± 27.5 | - 6.0 ± 15.4 | 0.02 |
| 3 months | 37 | 133.1 ± 20.7 | 132.5 ± 24.9 | - 0.2 ± 13.2 | 0.82 |
| 6 months | 32 | 134.0 ± 22.0 | 129.0 ± 22.3 | - 3.4 ± 9.7 | 0.05 |
|  |  |  |  |  |  |
| **Group A (Coenzyme Q10 + Selenium)** | | | | | |
| 0 | 40 | 130.7 ± 28.2 | - | - | - |
| 14 days | 38 | 131.5 ± 25.8 | 135.5 ± 31.1 | + 4.2 ± 20.5 | 0.35 |
| 3 months | 36 | 131.3 ± 27.9 | 138.3 ± 25.7 | + 7.3 ± 18.7 | 0.08 |
| 6 months | 30 | 125.0 ± 22.0 | 126.6 ± 26.5 | + 1.5 ± 17.9 | 0.67 |
|  |  |  |  |  |  |
| **Group B (Resveratrol + TA-65)** | | | | | |
| 0 | 40 | 130.5 ± 22.2 | - | - | - |
| 14 days | 39 | 130.2 ± 22.4 | 139.1 ± 26.0 | + 7.2 ± 11.5 | 0.0007 |
| 3 months | 34 | 130.6 ± 23.0 | 148.0 ± 29.1 | + 13.6 ± 13.4 | <0.0001 |
| 6 months | 33 | 131.5 ± 23.1 | 123.8 ± 21.6 | - 5.4 ± 9.3 | 0.002 |

Values are mean ± S.D. LDL cholesterol levels are expressed in mg/dl.

LDL cholesterol changes refer to the corresponding baseline values and are tested with paired t test.

N and baseline values change at each interval because only subjects with both baseline and after-interval value are included in the analysis.

Subjects with inadequate intake of treatments are excluded from this analysis. At the 5th month treatment intake was discontinued in Group B.

**Supplementary Table 10** – LDL cholesterol shift tables for the 3 groups

| **Group C - Placebo** | | | | |
| --- | --- | --- | --- | --- |
|  | Baseline | | | |
|  | Normal | Borderline High | High | Total |
| **14 days** |  | | | |
| Normal | 12 | 9 | 0 | 21 |
| Borderline High | 3 | 10 | 1 | 14 |
| High | 1 | 0 | 2 | 3 |
| Total | 16 | 19 | 3 | 38 |
| Missing | 0 | 1 | 0 | 1 |
| **3 months** |  | | | |
| Normal | 11 | 5 | 0 | 16 |
| Borderline High | 4 | 13 | 0 | 17 |
| High | 0 | 1 | 3 | 4 |
| Total | 15 | 19 | 3 | 37 |
| Missing | 1 | 1 | 0 | 2 |
| **6 months** |  | | | |
| Normal | 13 | 6 | 0 | 19 |
| Borderline High | 3 | 11 | 2 | 16 |
| High | 0 | 0 | 1 | 1 |
| Total | 16 | 17 | 3 | 36 |
| Missing | 0 | 3 | 0 | 3 |
| **Group A – Coenzyme Q10 + Selenium** | | | | |
|  | Baseline | | | |
|  | Normal | Borderline High | High | Total |
| **14 days** |  | | | |
| Normal | 15 | 5 | 0 | 20 |
| Borderline High | 3 | 8 | 1 | 12 |
| High | 2 | 1 | 3 | 6 |
| Total | 20 | 14 | 4 | 38 |
| Missing | 1 | 0 | 1 | 2 |
| **3 months** |  | | | |
| Normal | 11 | 2 | 0 | 13 |
| Borderline High | 7 | 7 | 1 | 15 |
| High | 1 | 4 | 3 | 8 |
| Total | 19 | 13 | 4 | 36 |
| Missing | 2 | 1 | 1 | 4 |
| **6 months** |  | | | |
| Normal | 12 | 5 | 0 | 17 |
| Borderline High | 6 | 8 | 4 | 18 |
| High | 1 | 0 | 1 | 2 |
| Total | 19 | 13 | 5 | 37 |
| Missing | 2 | 1 | 0 | 3 |
| **Group B – Resveratrol + TA-65** | | | | |
|  | Baseline | | | |
|  | Normal | Borderline High | High | Total |
| **14 days** |  | | | |
| Normal | 12 | 2 | 0 | 14 |
| Borderline High | 5 | 10 | 1 | 16 |
| High | 2 | 4 | 3 | 9 |
| Total | 19 | 16 | 4 | 39 |
| Missing | 0 | 1 | 0 | 1 |
| **3 months** |  | | | |
| Normal | 10 | 0 | 0 | 10 |
| Borderline High | 5 | 6 | 1 | 12 |
| High | 1 | 8 | 3 | 12 |
| Total | 16 | 14 | 4 | 34 |
| Missing | 3 | 3 | 0 | 6 |
| **6 months** |  | | | |
| Normal | 14 | 5 | 1 | 20 |
| Borderline High | 1 | 9 | 2 | 12 |
| High | 0 | 0 | 1 | 1 |
| Total | 15 | 14 | 4 | 33 |
| Missing | 4 | 3 | 0 | 7 |

Normal < 130 mg/dL, Borderline High 130-159 mg/dL, High ≥ 160 mg/dL
